# Comparative Analysis of Clinical and Radiological Outcomes of Unilateral Biportal Endoscopic Transforaminal Lumbar Interbody Fusion (UBE-TLIF) With Dual-Direction Expandable Cages Versus Anterior Lumbar Interbody Fusion at L5/S1

**DOI:** 10.7759/cureus.99309

**Published:** 2025-12-15

**Authors:** Alhareth Maaya, Jin Hwa Eum

**Affiliations:** 1 Neurosurgery, Ain Al Khaleej Hospital, Al Ain, ARE

**Keywords:** lumbar-fusion, minimally invasive surgery, spinal-fusion, transforaminal lumbar interbody fusion (tlif), unilateral bi-portal endoscopy

## Abstract

Objectives

The main objective of this study is to compare clinical and radiological outcomes of unilateral biportal endoscopic transforaminal lumbar interbody fusion (UBE-TLIF) with dual-direction expandable cages, versus anterior lumbar interbody fusion (ALIF) at L5/S1.

Summary of literature review

Traditional ALIF and TLIF approaches have limitations. UBE-TLIF with dual-direction expandable cages represents an emerging, minimally invasive alternative with potential advantages in tissue preservation and anatomical restoration.

Materials and methods

Six patients underwent UBE-TLIF at L5/S1 using expandable titanium cages. Clinical outcomes were assessed using the Visual Analog Scale (VAS) and the Oswestry Disability Index (ODI). Radiological parameters included disc height and neural foraminal height. Results were compared with ALIF outcomes from 11 literature studies.

Results

VAS decreased from 7.7 ± 1 to 4 ± 2 (back pain) and 8.2 ± 0.9 to 3 ± 2.1 (leg pain). ODI improved from 59 ± 5.8 to 27.5 ± 15. Disc height increased from 5.6 ± 1.5 mm to 15.8 ± 0.6 mm, and foraminal height from 8.8 ± 4 mm to 16.8 ± 3.4 mm (p < 0.05). Mean operative time was 269.5 minutes, with blood loss of 152 ± 30 mL.

Conclusions

UBE-TLIF demonstrates outcomes comparable to ALIF, with the advantages of single-position surgery and minimal tissue disruption, representing a viable, minimally invasive alternative for L5/S1 degenerative pathology.

## Introduction

Lumbar spine degenerative diseases, such as degenerative disc disease (DDD), spondylolisthesis, and lumbar spinal stenosis, are prevalent worldwide, cause severe disability, and interfere with quality of life. These diseases affect the L5/S1 segment, the transition from the lumbar spine to the sacral region, and can lead to chronic lower back pain, radicular pain, and neurogenic claudication. These symptoms can make physical activity difficult and result in major disability. Surgical intervention is often necessary when conservative treatment fails to address the dysfunction [1]. Endoscopic minimally invasive surgery (MIS) techniques, such as transforaminal lumbar interbody fusion (TLIF) and anterior lumbar interbody fusion (ALIF), have emerged to minimize invasive impact and promote recovery processes [2]. TLIF involves direct visualization and nerve decompression, while ALIF is an anterior-only approach that allows the surgeon to access the disc space directly, without disrupting posterior musculature and ligaments [3]. Surgical interventions have become less common due to advancements in techniques over the past few years [4]. Conventional methods of LMH have high morbidity, blood loss, and long postoperative recovery intervals, whereas endoscopic MIS techniques, such as TLIF and ALIF, have become predominant in spinal fusion at the L5/S1 level [5].

Unilateral biportal endoscopic (UBE)-TLIF is a MIS technique that improves conventional TLIF through a biportal approach. This procedure involves using an endoscopic tube and operative instruments inserted through two small puncture spots, or portals [6]. This portal system provides better visualization and maneuverability in the surgical field, thus facilitating a safer and less destructive approach to achieving disc height restoration and appropriate interbody dual-cage implantation. Another landmark development in the UBE-TLIF technique is that the dual cages used for expansion are reversible in two directions [7]. These dual expandable cages can be located at the disc site and expanded in situ, enabling accurate disc height restoration and increased segmental stability - two factors that are vital for successful spinal fusion and postoperative spinal stability [8].

ALIF is a type of spinal fusion surgery that involves approaching the lumbar spine through an abdominal incision. This approach has multiple benefits over posterior techniques, such as enabling direct visualization of the disc space, more extensive disc removal, and better endplate preparation [9]. The anterior approach allows implantation of larger interbody dual cages that may more effectively engage the endplate surface of the vertebral bodies, possibly enhancing fusion. ALIF has been shown to significantly improve lumbar lordosis [10]. By working directly on the anterior column of the spine, ALIF can help restore sagittal balance. Additionally, by preserving posterior elements, ALIF reduces the risk of paraspinal muscle atrophy and postoperative pain, resulting in a faster recovery period [11].

Radiological outcomes are essential for evaluating the long-term success of spinal fusion. The main radiologic parameters include fusion, disc height preservation, and segmental lordosis reconstruction, typically assessed using computed tomography (CT) and magnetic resonance imaging (MRI) [12]. Although cases involving instrumented fusion and segmental constructs are rare, previous studies observed that UBE-TLIF with dual-direction expandable cages has generated favorable radiological results in terms of disc height restoration and segmental stability [13]. These dual-direction expandable cages provide better conformability, especially when expanded with less stress and load in the disc space. ALIF, being more direct in its approach, has also shown its ability to exert high fusion rates, especially in restoring lordosis, thereby contributing to prolonged spinal stability in the treatment of degenerative lumbar conditions [14].

UBE-TLIF can be considered an example of an MIS approach intended to avoid tissue damage and compromise of anatomy, while ALIF, through its direct anterior approach, offers significant and easy access to the disc space [15]. This study examines unilateral dual-portal endoscopic TLIF using dual-direction expandable cages to treat lumbar degenerative conditions. It aims to provide insights into this innovative technique's safety, feasibility, and early clinical outcomes, contributing to the growing evidence supporting the utility of expandable cages in minimally invasive lumbar fusion surgery, and includes a comparison between our work and the literature review.

## Materials and methods

This study employs a retrospective comparative analysis to evaluate and compare clinical and radiological outcomes between two groups of patients: those who underwent UBE-TLIF at the L5/S1 level, as performed by the author, and those who underwent ALIF at the same level, based on data from existing literature. The primary objective is to determine the efficacy and superiority of UBE-TLIF in treating degenerative lumbar spinal conditions. Patients who underwent UBE-TLIF at the L5/S1 level, performed by the author within the specified study period, were included, and the inclusion criteria comprised patients diagnosed with degenerative lumbar conditions, such as spondylolisthesis, disc herniation, or spinal stenosis, at the L5/S1 level. This study was approved by the local ethics research committee of the organization, and the IRB approval number was AKH/2024007. All patient information was kept confidential, and the EQUATOR guidelines were followed for the study.

The surgical procedure utilizes dual (biportal) endoscopy and expandable titanium TLIF cages (Dual-X TLIF, Amplify Surgical, Inc., CA, USA), which start at a height of 7 mm and can expand up to 17 mm. The width begins at 12 mm and expands to 21 mm, with a final length of 30 mm and options for lordosis angles of 0°, 8°, 12°, and 15°. Anesthesia is administered, and the patient is placed in a prone position. Two incisions are made, one serving as the working portal and the other as the endoscopic portal, with lateral fluoroscopic imaging confirming accuracy. The endoscopic approach allows excellent endplate preparation under direct vision. A unilateral laminotomy with bilateral decompression may be performed, followed by disc space preparation and trial insertion. The final implant is selected, and autograft material is introduced. The expandable cage is inserted carefully while avoiding nerve root interference and positioned under fluoroscopic guidance. Allograft material is inserted after cage expansion and locking, a surgical drain is placed, endoscopic equipment is removed, and percutaneous pedicle screws are placed.

Pain levels were assessed using the Visual Analog Scale (VAS) [16], and preoperative and postoperative VAS scores were compared to quantify pain reduction following surgery. Functional outcomes were evaluated using the Oswestry Disability Index (ODI) [17], and both preoperative and postoperative ODI scores were recorded to measure functional improvement. Radiological assessments focused on fusion rates based on established radiological criteria, and imaging studies, including X-rays and CT scans, were analyzed to determine whether solid fusion was achieved at the L5/S1 level. Changes in disc height at the L5/S1 level were measured by comparing preoperative and postoperative imaging studies to assess the effectiveness of each surgical approach in restoring normal anatomy. Segmental lordosis was evaluated through radiological measurements, and preoperative and postoperative changes in segmental lordosis were analyzed to determine the impact of each surgical approach on spinal alignment and sagittal balance.

**Figure 1:**
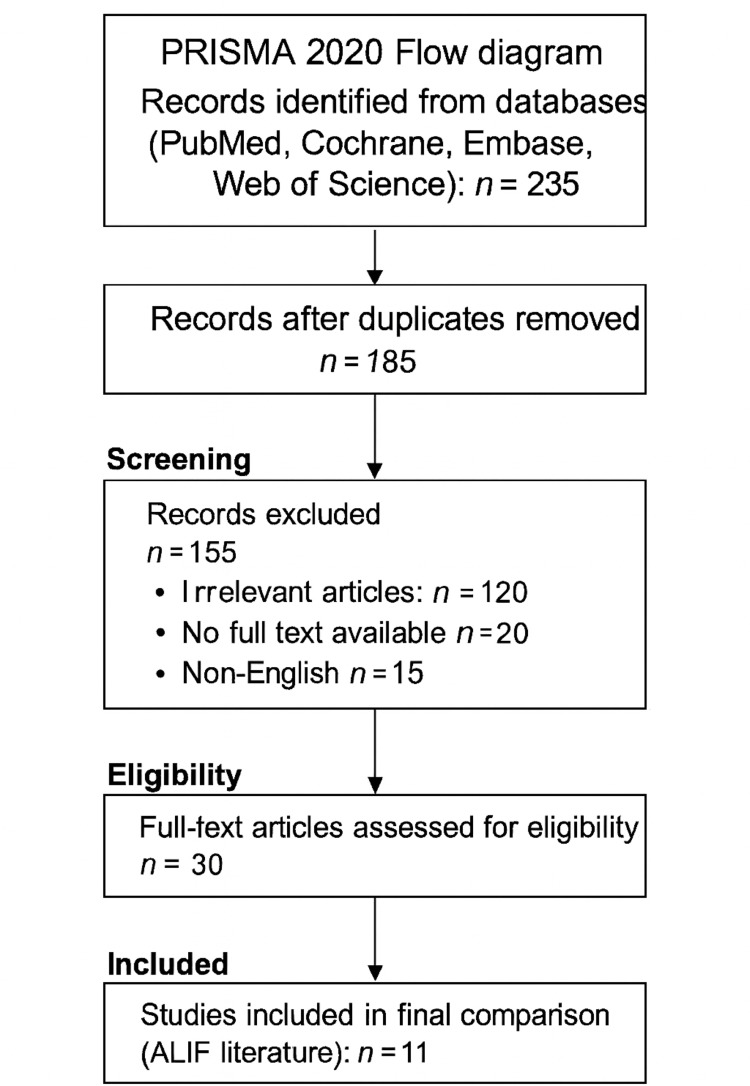
PRISMA 2020 flow diagram showing the study selection process for the review Adapted from PRISMA 2020 guidelines [18]

A systematic search of the literature, published from January 1, 2013, to December 31, 2023, was performed using the PubMed, Cochrane Library, Embase, and Web of Science databases. The Preferred Reporting Items for Systematic Reviews and Meta-Analyses (PRISMA) guidelines were adopted [18]. The following keywords were used for the comprehensive search: “anterior lumbar interbody fusion” and “ALIF L5/S1.” A total of 235 studies were identified through the database search, and 50 duplicates were removed. After title and abstract screening, 120 irrelevant studies, 20 articles without full text, and 15 non-English articles were excluded, leaving 30 potentially relevant studies. Of these, 19 did not match the inclusion criteria, and after full-text review, 11 studies remained and were selected for comparison with the findings of this study (Figure 1).

Descriptive statistics were calculated for continuous variables, such as VAS scores, ODI scores, fusion rates, disc height restoration, and segmental lordosis, including means, standard deviations, and confidence intervals. Inferential statistical tests were used to assess the significance of differences between the two groups, including t-tests or Mann-Whitney U tests for continuous variables, and chi-square tests for categorical variables. All statistical analyses were performed using appropriate statistical software, such as IBM SPSS Statistics for Windows, Version 29 (Released 2022; IBM Corp., Armonk, NY, USA) or R statistical software (R Foundation for Statistical Computing, Vienna, Austria), with a p-value <0.05 considered statistically significant.

## Results

Six patients underwent UBE-TLIF utilizing dual-direction expandable cages, and the preliminary results demonstrate promising outcomes, as the patients experienced significant pain reduction and improved functional status postoperatively. Table 1 shows that the patient cohort, comprising four males and two females with a mean age of 55 ± 9.4 years, predominantly underwent surgery at the L5-S1 segment. Diagnoses included four cases of grade 1 degenerative spondylolisthesis, one case of grade 2 degenerative spondylolisthesis, and one case of isthmic spondylolisthesis. The mean operation time was 269.5 minutes, with an estimated blood loss of 152 ± 30 mL, including the blood collected in the postoperative r/v drain. One patient experienced a complication of L5 partial palsy, which presented as severe numbness and paresthesia, and was managed conservatively, improving within a three-week period. The average hospital stay was 5.7 ± 1.7 days, with 50% of the patients having diabetes and 33% being smokers.

**Table 1:** Characteristics of the patient

Characteristic	Value
Age (yr)	55 ± 9.4
Sex, male:female	4:2
Operation segment	
L5-S1	6
Diagnosis	
Degenerative spondylolisthesis grade 1 [19]	4
Degenerative spondylolisthesis grade 2 [19]	1
Isthmus spondylolisthesis	1
Mean operation time (min)	269.5
Mean estimated blood loss (mL)	152 ± 30
Complication, L5 partial palsy	1
Hospital length of stay	5.7 ± 1.7
Diabetes (n, %)	3.50%
Smoker (n, %)	2.33%

Table 2 highlights significant clinical improvements post-surgery, with the VAS score for back pain decreasing from 7.7 ± 1 to 4 ± 2, and for leg pain from 8.2 ± 0.9 to 3 ± 2.1. Additionally, the ODI improved markedly, from 59 ± 5.8 preoperatively to 27.5 ± 15 postoperatively, reflecting enhanced functional status. 

**Table 2:** Clinical results VAS: Visual Analog Scale; ODI: Oswestry Disability Index

Variable	Preoperative	Postoperative
VAS back [16]	7.7 ± 1	4 ± 2
VAS leg [16]	8.2 ± 0.9	3 ± 2.1
ODI [17]	59 ± 5.8	27.5 ± 15

Table 3 represents the radiographic results, which show that disc height at the operative segment improved significantly from 5.6 ± 1.5 mm to 15.8 ± 0.6 mm, and neural foraminal height increased from 8.8 ± 4 mm to 16.8 ± 3.4 mm, both with p < 0.05, demonstrating the effective restoration of lumbar spine anatomy and successful spinal fusion. These outcomes suggest that UBE-TLIF, using dual-direction expandable cages, is a promising technique for treating spondylolisthesis, with clinical and radiographic evidence supporting its efficacy. 

**Table 3:** Radiographic results

Variable	Preoperative	Postoperative
Disc height of operative segment (mm)	5.6 ± 1.5	15.8 ± 0.6
Neural foraminal height (mm)	8.8 ± 4	16.8 ± 3.4

**Figure 2:**
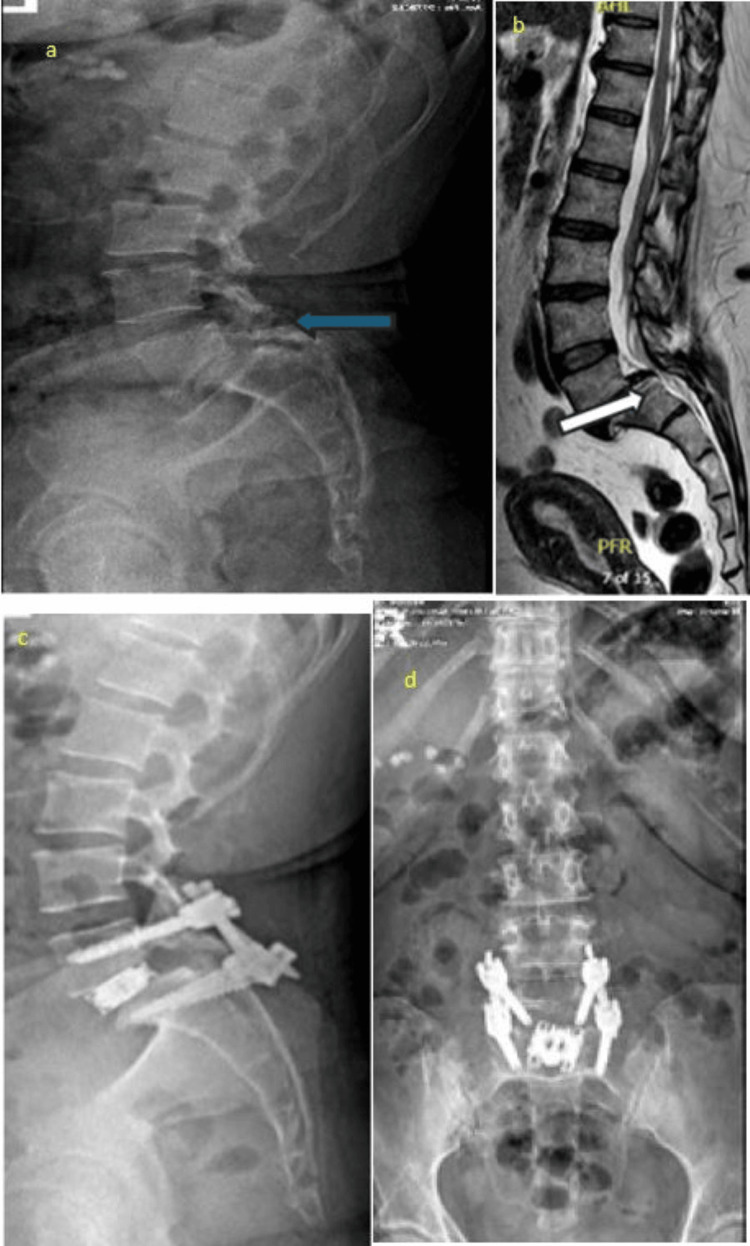
Grade 2 spondylolisthesis a) A 56-year-old female presented with low back pain and left lower-extremity symptoms. Unilateral biportal endoscopic transforaminal lumbar interbody fusion (UBE-TLIF), with unilateral laminotomy and bilateral decompression, was performed using a dual-direction expandable titanium cage via a left-sided approach. Preoperative lateral X-ray shows lower lumbar degenerative changes and grade 2 L5-S1 spondylolisthesis, with collapse of the intervertebral disc space. b) Preoperative sagittal T2-weighted magnetic resonance imaging (MRI) of the lumbosacral spine showing grade 2 spondylolisthesis and associated disc degeneration at the L5-S1 level (white arrow). c) Postoperative lateral X-ray showing the dual-direction expandable cage inserted at the L5-S1 disc space. The intervertebral space is expanded following cage insertion, demonstrating excellent reduction, improved alignment, and restoration of both disc height and foraminal height. Pedicle screws are in place. d) Postoperative anteroposterior (AP) X-ray showing the dual-direction expandable cage in situ, with a large footprint providing stable interbody support. Pedicle screws are symmetrically placed, confirming satisfactory alignment and fixation.

The follow-up period for the patients ranged from 44 to 54 weeks postoperatively. During this time, regular clinical assessments and radiographic evaluations were conducted to monitor progress and assess the stability of the surgical outcomes. The lateral lumbosacral X-ray illustrates the patient’s initial condition, showing grade 2 spondylolisthesis, which serves as a baseline for assessing the severity of vertebral slippage prior to surgical intervention (Figure 2a). 

Figure 2c represents a follow-up lateral lumbosacral X-ray, revealing remarkable improvement post-surgery. The spondylolisthesis appears significantly reduced, indicating successful realignment of the affected vertebrae. The expandable cage is clearly visible, demonstrating effective restoration of disc height and neural foraminal height.

The anterior-posterior X-ray provides a frontal view of the spine post-surgery and showcases the placement of the dual-direction expandable cage, highlighting its substantial footprint within the intervertebral space. This confirms the stability and proper positioning of the implant, as shown in Figure 2c.

Figure 2b presents a lateral T2 MRI, offering a different perspective on the patient’s condition. Like Figure 2a, this image shows grade 2 spondylolisthesis preoperatively, providing additional insight into the extent of vertebral slippage and the underlying pathology. 

**Figure 3:**
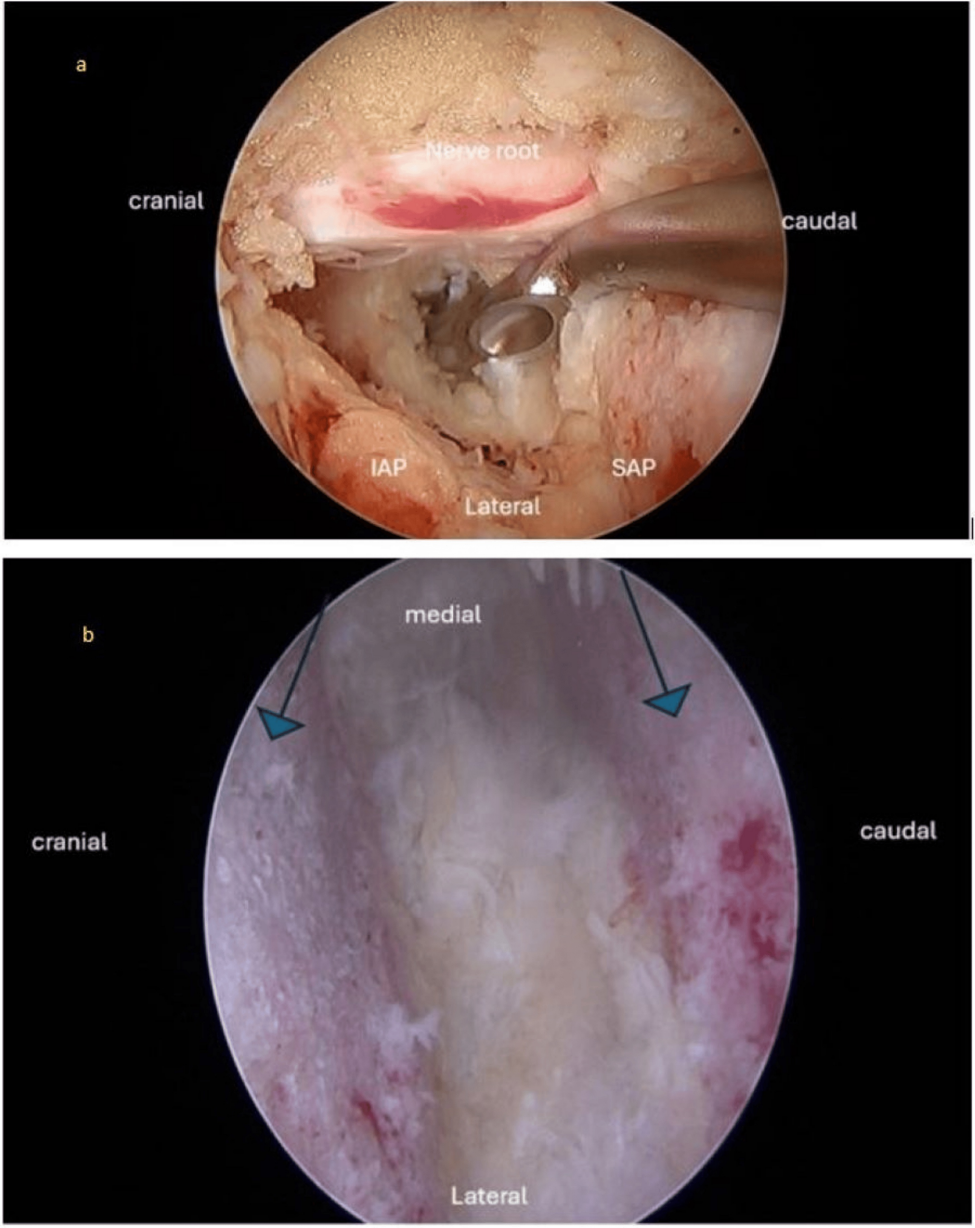
Intraoperative endoscopic a) Intraoperative endoscopic photograph showing the exposed dura and traversing nerve root, following completion of unilateral laminotomy and bilateral decompression. The decompressed nerve root is clearly visualized. b) Intraoperative endoscopic photograph showing the intervertebral disc space after complete discectomy and meticulous endplate preparation. Blue arrows indicate excellent endplate preparation, visualized under the endoscopic view. SAP, Superior Articular Process; IAP, Inferior Articular Process

Figure 3a depicts an intraoperative endoscopic photograph, revealing the dura and the traversing nerve root following the completion of unilateral laminotomy and bilateral decompression. This image provides a detailed view of the surgical field, showcasing the careful surgical technique employed to decompress neural structures. The exposure of the dura and nerve root highlights the precision and thoroughness of the surgical approach, crucial for relieving neural compression and restoring spinal function. 

Figure 3b illustrates an intraoperative endoscopic photograph, displaying the intervertebral disc space following complete discectomy and meticulous endplate preparation, including the removal of the cartilaginous endplate to facilitate fusion. This image provides a clear visualization of the surgical site, demonstrating the thoroughness of the disc removal process and the preparation of the vertebral endplates. The absence of disc material and cartilaginous tissue indicates the readiness of the space for fusion and instrumentation, emphasizing the precision required for successful spinal stability. 

## Discussion

The results of this study indicate that UBE-TLIF, using dual-direction expandable cages, offers significant clinical and radiological benefits for patients with degenerative lumbar spine conditions at the L5/S1 level. This minimally invasive technique has shown promising outcomes in terms of pain reduction, functional improvement, and anatomical restoration. The clinical results demonstrated substantial improvements in both pain and functional status postoperatively. The decrease in VAS scores for back and leg pain highlights the effectiveness of UBE-TLIF in alleviating pain symptoms. Additionally, the significant reduction in the ODI reflects enhanced functional capabilities and overall quality of life for patients. Radiologically, UBE-TLIF has successfully restored disc height and increased neural foraminal height, as evidenced by preoperative and postoperative measurements, suggesting that the dual-direction expandable cages effectively restore lumbar spine anatomy and contribute to segmental stability, which is crucial for successful spinal fusion.

UBE-TLIF offers clinical and radiological outcomes comparable to those of ALIF. Studies have shown similar successful fusion rates, pain relief, and functional improvement, making it a viable alternative to ALIF. This study aligns with Berjano et al. [20] in demonstrating the efficacy of ALIF at L5-S1, especially in revision spine surgeries. Both studies highlight the reliable outcomes of ALIF in achieving solid fusion and pain relief. However, Berjano et al. [20] emphasized the additional stability provided by posterior lumbosacral instrumentation, which was not a focal point in this research. This suggests that, while ALIF alone is effective, combining it with posterior support may enhance outcomes in complex cases.

Surgical complications remain a significant concern in lumbar interbody fusion procedures, with diverse approaches yielding varying complication rates. Cui et al. [21] identified complications in 15.3% of patients who underwent lateral anterior lumbar interbody fusion (LaLIF), with temporary psoas injury and sympathetic chain injury among the common complications encountered. These findings underscore the importance of meticulous surgical technique and perioperative management to mitigate adverse events.

Further studies evaluating ALIF outcomes emphasize the need for careful consideration of complication risks and benefits. Demirel et al. [22] observed a 16.0% complication rate in ALIF surgeries, with a remarkably low non-union rate of 2.5%, suggesting favorable outcomes despite potential perioperative challenges. Conversely, Epstein [23] highlighted the spectrum of major complications associated with ALIF - including sympathectomy, vascular injuries, and bowel perforations - emphasizing the need for vigilant intraoperative monitoring and surgical expertise. Feeley et al. [24] conducted a systematic review showing that the transperitoneal approach in ALIF procedures is associated with higher rates of retrograde ejaculation and overall complications, indicating that careful approach selection is essential to minimizing adverse outcomes.

UBE-TLIF is performed in a single prone position, eliminating the need to reposition the patient. ALIF often requires two positions: supine for cage insertion and prone for posterior instrumentation. This streamlining reduces operative time and potential repositioning-related complications. Tye et al. [25] found that ALIF outperforms TLIF with respect to segmental and regional radiographic outcomes and patient-reported measures. The current results corroborate these findings, showing superior radiographic and clinical improvements with ALIF, and reinforcing the preference for the anterior approach in L5-S1 isthmic spondylolisthesis.

The prone position used in UBE-TLIF is more anatomical and favorable for accessing the spine compared with the lateral position used in some ALIF cases. Dowlati et al. [26] identified the vulnerability of the L5 nerve root during ALIF procedures. Although this study did not specifically address nerve root vulnerability, the low incidence of nerve-related complications in our cohort may be attributed to meticulous technique, highlighting the importance of careful procedural planning.

ALIF typically necessitates the involvement of an access surgeon, such as a vascular surgeon, to safely navigate the anterior spine. This requirement adds procedural complexity. Edwards et al. [27] compared ALIF and TLIF radiographic outcomes at L5-S1 and demonstrated superior disc height restoration and sagittal balance correction with ALIF, findings consistent with the present study. This supports the radiographic advantages of the anterior approach.

ALIF carries higher risks of vascular injury, retrograde ejaculation, and complications related to anterior access, particularly in obese patients or those with prior abdominal surgeries. Fleege et al. [28] reviewed ALIF and PLIF for low-grade isthmic spondylolisthesis, highlighting effective fusion rates for both approaches but emphasizing the minimally invasive nature and faster recovery of ALIF. This supports the potential advantages of ALIF in terms of recovery and surgical invasiveness.

UBE-TLIF is particularly advantageous for obese patients or those with prior abdominal surgery, as it avoids anterior access entirely. Jia et al. [29] systematically reviewed axial interbody arthrodesis at L5-S1, noting its efficacy and safety. The present outcomes align with these findings, demonstrating high fusion and low complication rates with ALIF. This parallel reinforces ALIF’s reliability in L5-S1 fusion.

The use of expandable cages in UBE-TLIF allows insertion of larger cages through minimal incisions, supporting better spinal alignment and potentially superior fusion outcomes. Lightsey et al. [30] demonstrated superior clinical and radiographic outcomes with ALIF compared to TLIF. This study similarly shows that ALIF improves both patient-reported and radiographic parameters, suggesting that ALIF should be considered the preferred approach for achieving optimal outcomes at L5-S1.

Expandable cages with a lordotic angle help restore natural spinal curvature, contributing to biomechanical alignment and postoperative spinal health. Mobbs et al. [31] described ALIF’s technical aspects and outcomes at L5-S1, highlighting its effectiveness in achieving fusion and symptom relief. The present study supports these findings by showing similar success and positive patient outcomes.

UBE-TLIF utilizes tiny incisions with minimal muscle dissection. This minimally invasive approach reduces postoperative pain, promotes faster recovery, and minimizes risks such as muscle atrophy or scarring. Phan et al. [32] conducted a meta-analysis on ALIF for recurrent disc herniations, showing high success and low complication rates. These observations align with the present findings. Sebastian et al. [33] compared ALIF and TLIF for low-grade isthmic spondylolisthesis and concluded that ALIF offered better outcomes; our study similarly shows superior clinical and radiographic results with ALIF.

Uribe et al. [34] reported immediate reciprocal changes at adjacent levels following single-level ALIF. Although adjacent segment changes were not the focus of this study, minimal adverse effects were observed, consistent with Uribe et al. [34]. This supports the stability of ALIF without compromising adjacent segments.

Overall, comparing ALIF and UBE-TLIF reveals important differences. ALIF improves biomechanical stability and restores disc height, whereas UBE-TLIF offers minimal invasiveness, reduced operative time, and faster recovery. Expandable cages enhance both techniques by improving fusion potential and restoring lordosis. UBE-TLIF provides several advantages, including single-position surgery, better anatomical access, lower complication rates, and superior suitability for obese patients or those with previous abdominal surgeries. Although the preliminary results are promising, the small sample size and limited follow-up require validation through larger studies. A direct comparative study between UBE-TLIF and ALIF would provide more robust evidence.

## Conclusions

The study concludes that UBE-TLIF is a minimally invasive procedure that reduces pain, improves functional status, and restores lumbar spine anatomy. Compared with ALIF, UBE-TLIF offers single-position surgery, better anatomical alignment, and lower complication rates. However, further research is needed to validate the results and establish the long-term efficacy of UBE-TLIF. The findings affirm that UBE-TLIF offers similar clinical and radiological outcomes to ALIF, including pain reduction, functional improvement, and anatomical restoration. Clinicians can now consider UBE-TLIF as a viable alternative technique, potentially expanding treatment options for patients with degenerative lumbar spine conditions.
